# Analysing the Costs of Integrated Care: A Case on Model Selection for Chronic Care Purposes

**DOI:** 10.5334/ijic.2422

**Published:** 2016-08-19

**Authors:** Marc Carreras, Inma Sánchez-Pérez, Pere Ibern, Jordi Coderch, José María Inoriza

**Affiliations:** 1 Research group on health services and health outcomes (GRESSIRES) – Serveis de Salut Integrats Baix Empordà (SSIBE) and Universitat de Girona, ES; 2 Research group on health services and health outcomes (GRESSIRES) – Serveis de Salut Integrats Baix Empordà (SSIBE), ES; 3 Centre for research in health and economics (CRES) – Universitat Pompeu Fabra, ES

**Keywords:** costs and cost analysis, delivery of health care, integrated, risk assessment

## Abstract

**Background:**

The objective of this study is to investigate whether the algorithm proposed by Manning and Mullahy, a consolidated health economics procedure, can also be used to estimate individual costs for different groups of healthcare services in the context of integrated care.

**Methods:**

A cross-sectional study focused on the population of the Baix Empordà (Catalonia-Spain) for the year 2012 (N = 92,498 individuals). A set of individual cost models as a function of sex, age and morbidity burden were adjusted and individual healthcare costs were calculated using a retrospective full-costing system. The individual morbidity burden was inferred using the Clinical Risk Groups (CRG) patient classification system.

**Results:**

Depending on the characteristics of the data, and according to the algorithm criteria, the choice of model was a linear model on the log of costs or a generalized linear model with a log link. We checked for goodness of fit, accuracy, linear structure and heteroscedasticity for the models obtained.

**Conclusion:**

The proposed algorithm identified a set of suitable cost models for the distinct groups of services integrated care entails. The individual morbidity burden was found to be indispensable when allocating appropriate resources to targeted individuals.

## Introduction

Demographic and epidemiological changes are two key driving forces behind integrated care. In recent decades, public health services in modern societies have been facing huge challenges, namely in the form of tight budgets and aging populations with an increasing number of chronic diseases. Many of the distinctive features of integrated care models have been extensively discussed in parallel to its progressive implementation; however, the economic issues related to integrated care remain relatively unexplored. A recent editorial by Evers and Paulus published in the International Journal of Integrated Care, stated that the health economics of integrated care is “still in its infancy” and called for the development of new methods tailored to the special characteristics of integrated care [1]. Moreover, the authors highlighted several of the challenges previously identified and discussed by a panel of experts at the International Foundation of Integrated Care’s (IFIC) 14th International Conference on Integrated Care in Brussels, 2014. These challenges included assessing the quality of the literature currently available on integrated care, providing outcome measurements as well as valuing and determining integrated care costs.

Different situations may require either an estimation of individual costs or one of episode care costs. For example, when a capitation based payment agreement regulates the financial relationship between a healthcare provider and a healthcare financing organization. In this case, information about the burden of chronic diseases together with the costs of healthcare services received by individuals is essential for a health planner to be able to allocate resources according to population needs. Moreover, healthcare providers involved in chronic care strategies would most likely need to identify both target patients, who need tailored care, as well as patients with potentially high costs. A second common case is related to measuring costs when carrying out economic evaluations. Beyond traditional care schemes, there is little evidence on costs related to the healthcare episodes involved in the new forms of care delivery, e.g. integrated nursing home care compared to traditional care [1,2,3]. Therefore, an accurate estimation of costs adapted to the creative features of integrated care processes may be required.

Health economics literature includes a large number of contributions which deal with analysing both, individual health expenditure and resource consumption, e.g. [4,5,6,7,8,9]. In particular, since the early development of the RAND Health Insurance Experiment (HIE) [10], a large body of methodological papers has identified the main issues related to the econometric analysis of health expenditure. Within this framework, the most common difficulties include excess of zeros in the data (caused by the inclusion of non-users of health services in the samples), skewness and heteroscedasticity. A good variety of estimation approaches have provided solutions to these problems. Among others, linear models (LM) fitted to the log expenditures or generalized linear models (GLM) with a log link are probably the most prevalent [11,12]. In order to provide some guidance throughout models, Manning and Mullahy provided, what would later become a popular method among analysts, a straightforward algorithm which fits a reasonable model in a small number of steps [6]. The main advantage of using this algorithm is that it considerably reduces the amount of time dedicated to econometric fine-tuning. The original work was cited by over 200 articles, which addressed wide-ranging issues concerning healthcare resource use and costs. In general, the individual cost of patients, treatments or services is defined as a dependent variable related to some set of independent variables or risk factors. Recent work using the algorithm includes examples of cost-effectiveness, cost and quality of life, analysis of expenditure or simply cost analysis models, covering a wide variety of topics from acute injuries to infectious or chronic diseases. However, the algorithm has yet to be proven in an integrated care context, including complex care processes and heterogeneous combinations of health services. Although previous research work has analyzed cost models in an integrated care context [13,14,15,16], none, at least to our knowledge, have used the algorithm proposed by Manning and Mullahy [6].

Our motivation for obtaining the costs of integrated care comes from the necessity for efficient chronic care strategies. Throughout this paper we take the perspective of an integrated healthcare provider involved in the design of patient centered strategies, which also implies allocating resources. The objective of the study is to investigate whether the algorithm proposed by Manning and Mullahy can be used to estimate individual costs for different groups of healthcare services, as a function of sex, age and morbidity burden, in a context of integrated care services.

## Methods

A cross-sectional study was carried out using the information collected during the year 2012. Data were provided by Serveis de Salut Integrats Baix Empordà (SSIBE), a fully integrated organisation responsible for the delivery of public health services in the county of the Baix Empordà, located in the Province of Girona (Spain). SSIBE care services include primary care, acute-care hospitalization, ambulatory care, emergencies and long-term residential care. SSIBE run an information system, which includes individual-level clinical records, activity logs and resource consumption details. The study focused on the Baix Empordà population covered by SSIBE: N = 92,498 individuals of whom 76,360 were health service users with positive costs. The total cost of the healthcare services provided by SSIBE during 2012 amounted to 68,943,180 Euros.

### Individual costs

**Figure 1:**
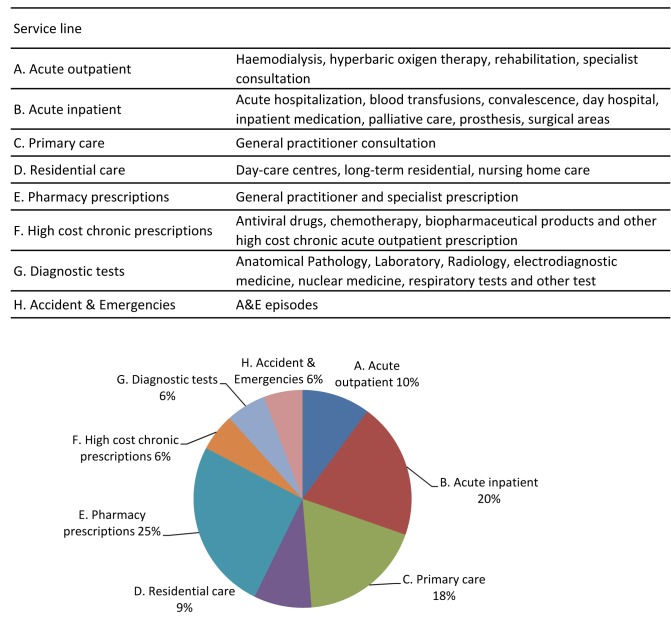
SSIBE healthcare services.

Healthcare costs were obtained using a retrospective full-costing system. Costs were calculated for each individual in the population from the sequence of healthcare episodes and pharmacy prescriptions they received in 2012. A detailed description of the healthcare services provided by SSIBE is shown in Figure 1. The SSIBE costing system meets the requirements established by the working group on cost accounting for the Spanish network of hospital costs (RECH) [17,18].

The choice of a healthcare provider viewpoint implies that indirect or community costs, beyond formal care, were not included in the model. For example, informal care costs or productivity losses.

### Morbidity burden

We included an assessment of the individual morbidity burden using the Clinical Risk Groups patient classification system (3M™CRG) software version 1.9.1 [19,20,21]. The clinical category and the health status of each individual were obtained from the 1,410,351 ICD-9-CM activity codes (diagnostics and procedures) and the 1,563,169 pharmaceutical prescriptions (coded by ATC classification) generated through health service usage in 2012. Within the CRG model, individuals were classified into single, mutually exclusive and exhaustive categories according to their clinical records. The standard CRG classification system contains nine categories: (1) Healthy, (2) History of significant acute disease, (3) Single minor chronic disease, (4) Minor chronic disease in multiple organ systems, (5) Single dominant or moderate chronic disease, (6) Significant chronic disease in multiple organ system, (7) Dominant chronic disease in three or more organ systems, (8) Dominant and metastatic malignancies and (9) Catastrophic conditions. A summary of the Baix Empordà population morbidity is presented in the Table 1 [22]. Data were collected on 31/12/2012. The information has been sorted by health status and severity level (the ACRG3 level using the Clinical Risk Groups terminology).

**Table 1:** Percent of population by morbidity burden.

	Severity level							
Health Status	0	1	2	3	4	5	6	Total

1. Healthy	32.65%	16.95%	0.26%		0.31%	1.50%		51.69%*
2. History Of Significant Acute Disease	4.65%		0.54%		0.16%	1.31%		6.66%**
3. Single Minor Chronic Disease	–	6.67%	2.10%					8.77%
4. Minor Chronic Disease In Multiple Organ Systems	–	1.07%	0.17%	1.01%	0.48%			2.73%
5. Single Dominant Or Moderate Chronic Disease	–	9.16%	2.79%	0.75%	0.05%	0.09%	0.01%	12.85%
6. Significant Chronic Disease In Multiple Organ Systems	–	6.75%	3.12%	2.52%	1.73%	0.64%	0.09%	14.86%
7. Dominant Chronic Disease In Three Or More Organ Systems	–	0.29%	0.26%	0.60%	0.16%	0.12%	0.03%	1.47%
8. Dominant, Metastatic, And Complicated Malignancies	–	0.06%	0.19%	0.23%	0.15%	0.03%		0.66%
9. Catastrophic Conditions	–	0.02%	0.10%	0.06%	0.06%	0.03%	0.05%	0.32%

Cross-sectional data at 31/12/2012. N = 92,498 individuals. Clinical Risk Groups classification system. ACRG3: Health Status & severity level. * Healthy 1.0 Healthy 1.1 Healthy Non-User 1.2 Delivery without Other Significant Illness 1.4 Pregnancy without Delivery without Other Significant Illness 1.5 Evidence of Significant Chronic or Acute Diagnosis without Other Significant Illness ** History Of Significant Acute Disease 2.0 History Of Significant Acute Disease 2.2 Delivery with History of Significant Acute Illness 2.4 Pregnancy without Delivery with History of Significant Acute Illness 2.5 Evidence of Significant Chronic or Acute Diagnosis with History of Significant Acute Illness

We included the costs of the healthcare services described in Figure 1, with the exception of Residential care (line D). In this case, the information system registered a reduced number of ICD codes for users of Residential care living in nursing homes. This problem is attributable to administrative causes, but resulted in an underestimation of morbidity.

### Statistical analysis

We fitted an estimator of positive costs for healthcare service lines described in Figure 1, with the exception of Residential care. We added an additional model for the total cost of healthcare services. The dependent variable was the individual cost per year. Independent variables were age, sex, and health status (ACRG3 level). All variables were considered categorical with the exception of age, which was introduced as a continuous variable. To analyse the coefficients, we set a significance level of 0.05.

According the Manning-Mullahy criteria, we chose between two alternative estimators:

1. Linear model on the log costs: *logY_i_* = *α*+*βX_i_* +*ε_i_*
2. Generalized linear model with a log link: *Y_i_* = *e*^*α*+*βX_i_*+*ε_i_*^

**Figure 2:**
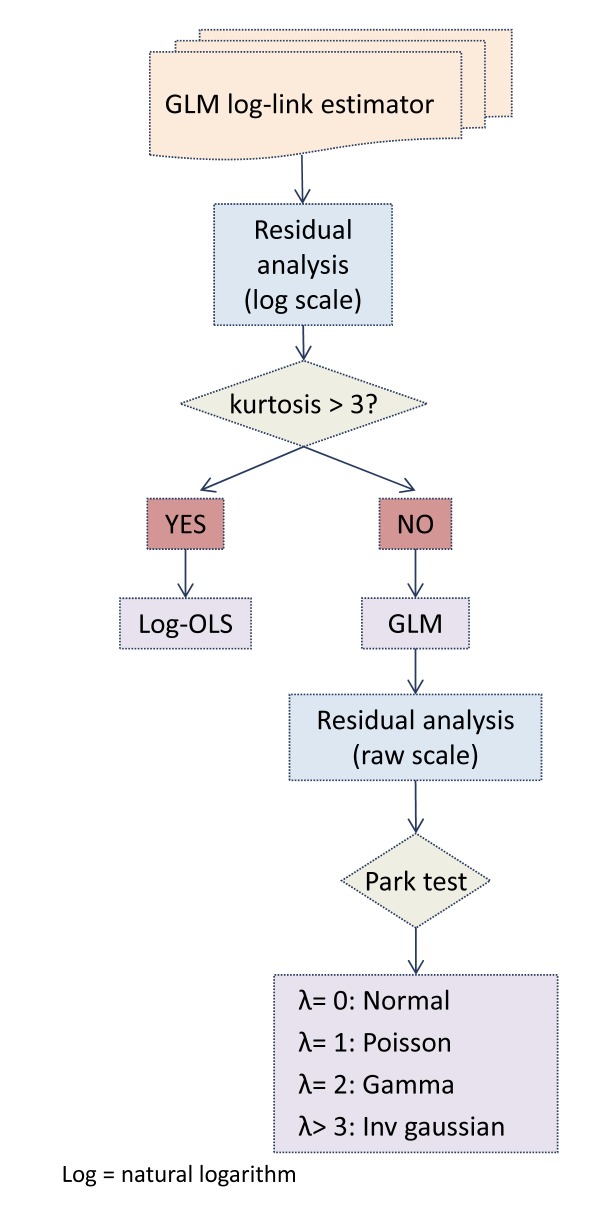
Manning – Mullahy Algorithm.

Following the recommended steps (see Figure 2) we started with a GLM-Gamma model with log-link. Once a preliminary model was available, we proceeded to the analysis of the residuals:

- If the log-scale residuals were not heavy-tailed (kurtosis = <3), we maintained the GLM class. Then, using the Park test, we searched for an accurate variance function. Park tests identify the optimal function for the variance of the residuals (in the observed raw-scale). From lowest to highest degree of dispersion, candidate functions are: Gaussian, Poisson, Gamma or inverse Gaussian (Wald). The Park test has been widely discussed in econometric literature since it was first published [23].
- Conversely, if the log-scale residuals were heavy-tailed (kurtosis clearly above 3), we fitted a linear model on the log costs. According to the literature, if the log-scale residuals are heavy-tailed, models within the GLM class result in a biased estimator [6,8].

It is important to note that the cut-off point for the decision algorithm, a kurtosis coefficient “about 3” in words of the authors, allows for some flexibility. For example, a GLM model showing a slightly higher kurtosis can be accepted if the precision statistics exhibit reasonable values.

We included a set of tests for verifying the robustness of the results: A modified Hosmer-Lemeshow test, the Mean Absolute Percentage Error (MAPE), the Median Absolute Percentage Error (MEDAPE), and the Pregibon Link Test [24,25,26,27].

The modified Hosmer-Lemeshow test is based on an F-test of whether the mean of residuals throughout all groups of deciles are not significantly different from zero. The idea is to detect systematic patterns of bias [24].

The Mean Absolute Percentage Error (MAPE) and the Median Absolute Percentage Error (MEDAPE) accounted for accuracy and are based on the distribution of absolute errors: MAPE = (Σ|*PE*|)/*n*, MEDAPE = *median* (|*PE*|) [25]. Since both measures are based on percentages, they account for the relative size of the error and therefore are not scale-dependent.

The Pregibon Link Test examined the adequacy of the link function specification assumed in the GLM models [28]. In accordance with the algorithm, we restricted the link function to the natural logarithm.

Furthermore, if the output of the algorithm was a linear model on the log costs, we tested for heteroscedasticity using the Breusch-Pagan test [28]. For heteroscedastic log-ols models, we readjusted the coefficients using a secondary variance model, based on the log of the squared residuals [5,29].

All methods, estimation procedures and statistical tests described are embedded or programmable in the main statistical software packages. Throughout this work we used the R 3.1.2 software version [30].

### Ethics

The study protocol was approved by the Clinical Research Committee of SSIBE. Given the methodology of the study, based on a retrospective review of clinical and administrative records, no informed consent was requested. Data management was conducted anonymously by members of the SSIBE staff.

## Results

### Model Choice

Table 2 summarises the results of the algorithm for the healthcare service models included in the analysis. The first column shows the number of individuals with positive costs for each service line. The second and the third columns include the kurtosis coefficient for the log-scale residuals and the resulting model (a GLM or linear model on the log of costs, respectively). When the result was a GLM, the fourth and fifth columns include the Park test and the corresponding variance function respectively. Otherwise, the fourth and fifth columns appear empty. For all groups of services, except for Pharmacy prescriptions and Total healthcare cost, the kurtosis coefficient was about 3 or less and therefore the recommended option resulted in a GLM. For all these models, according to the Park test, the variance of the residuals was described by the Gamma distribution, with the exception of the High cost chronic prescriptions, which resulted in a Poisson distribution. For Pharmacy prescriptions and Total healthcare cost, the kurtosis coefficient was clearly above 3 and, consequently, following the algorithm recommendations we adjusted a linear model on the log of costs.

**Table 2:** Algorithm results: Model choice for individuals with positive costs.

Service line	N	kurtosis	Model	Park	Distribution	Hosmer-Lemeshow (p-value)	MAPE/100	MEDAPE/100	Pregibon link (p-value)	Breusch Pagan (p-value)

1. Acute Outpatient	33,949	3.06	GLM	2.21	Gamma	0.09 (0.999)	1.43	0.68	–0.001 (0.926)	–
2. Acute inpatient	7,403	2.7S	GLM	2.17	Gamma	0.15 (0.999)	4.70	0.75	–0.002 (0.980)	–
3. Primary care	72,514	2.91	GLM	1.79	Gamma	56.33 (<0.001)	1.07	0.51	–0.117 (<0.001)	–
4. Pharmacy prescriptions	61,682	3.35	Log-ols	–	–	23.39 (<0.001)	0.39	0.19	–	1,949.39 (<0.001)
5. High cost chronic prescriptions	947	2.31	GLM	1.00	Poisson	1.29 (0.233)	20.20	1.03	0.005 (0.855)	–
6. Diagnostic tests	42,273	2.69	GLM	2.08	Gamma	2.22 (0.014)	1.66	0.76	–0.022 (0.162)	–
7. Accident & Emergencies	19,949	3.07	GLM	1.94	Gamma	1.02 (0.422)	2.02	0.73	–0.012 (0.526)	–
8. Total healthcare cost	76,360	3.40	Log-ols	–	–	46.12 (<0.001)	0.14	0.10	–	1,227.20 (<0.001)

### Robustness

Summary statistics are shown in the right half of the Table 2. For all the GLM models, the F-test was not significant with the exception of Primary Care. For log-ols models the F-test resulted significant as well. Fitted models included a similar level of error, with the exception of Acute inpatient care (MAPE = 4.70) and High cost chronic prescriptions (MAPE = 20.20). However, for these models the presence of outliers substantially inflated the result of the MAPE, as indicated by the low values of the MEDAPE: 0.75 and 1.03, respectively. Examining the Pregibon Link Test results, the log specification was correct for all but Primary care, of the GLM models obtained. Finally, according to the Breusch-Pagan test, log-ols models resulted heteroscedastic, mainly caused by morbidity variables.

### Individual profile and resource consumption

**Figure 3:**
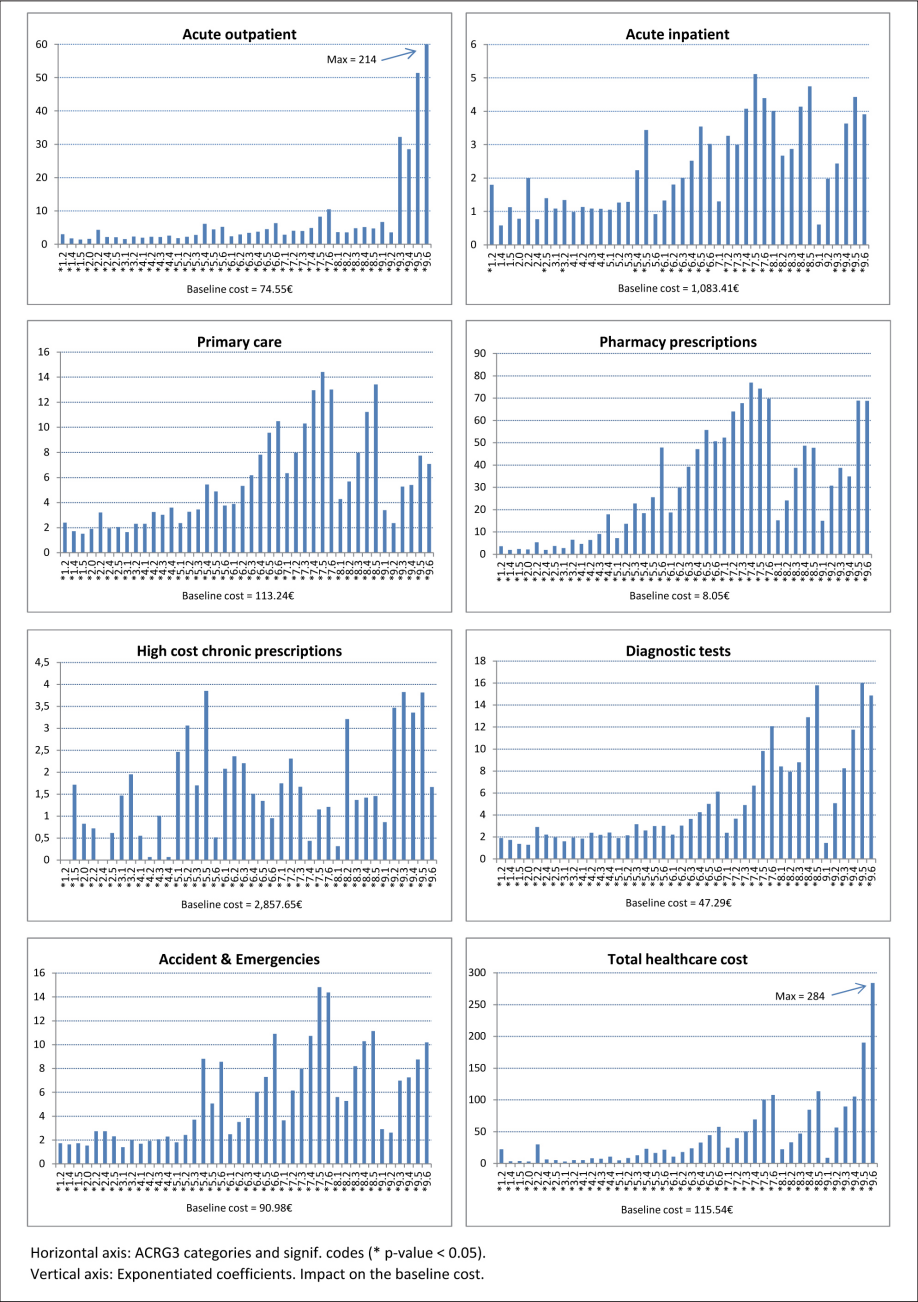
Impact of morbidity on resource consumption.

Figure 3 shows the morbidity coefficients {e^{\hat \beta }} together with the level of significance. Since our analysis relies on log models, the coefficients {e^{\hat \beta }} from categorical variables should be interpreted as the incremental impact in costs over the baseline, 1.0 Healthy for morbidity and female for sex.

We decided to show demographic coefficients separately for clarity, but at the same time for the different scale of impact in costs (see Table 3). In general, demographic variables showed a lower level of influence. For example, the maximum impact for morbidity variables was for 9.6 Catastrophic Conditions – Level 6 patients, which multiplies the cost of the baseline morbidity category 284 times. Whereas, the maximum impact for demographic variables was for sex and for patients having High cost chronic prescriptions. In this case, baseline costs for men increased by 1.21. Moreover, demographic variables showed a lower and more heterogeneous level of significance.

**Table 3:** Impact of Age and sex in resource consumption.

Service line	Age	Sex (male)

1. Acute Outpatient	1.0012*	0.9786
2. Acute Inpatient	1.0014	0.9641
3. Primary care	0.9912*	0.8925*
4. Pharmacy prescriptions	1.0126*	0.8503*
5. High cost chronic prescriptions	0.9881*	1.2182*
6. Diagnostic tests	0.9993	0.8655*
7. Accident & Emergencies	0.9973*	0.9952
8. Total healthcare cost	0.9952*	0.8661*

* p-value < 0.05.

Different patient profiles resulted in a different impact on the health system. Figure 3 (left to right) shows how resource consumption – and costs – increased progressively, i.e. from the healthiest to the unhealthiest conditions. Such general behaviour was expressed in different degrees for most of the healthcare service lines: Patients with three or more chronic conditions (CRG health status 7.X) or classified as Catastrophic Conditions had a substantial impact in costs. The exception was High cost chronic prescriptions, which showed a less progressive behaviour caused by patients classified in low complexity CRG categories being prescribed costly biological drugs. For example, patients taking anti-rheumatic drugs could be classified as 3.x Single Minor Chronic Disease or patients taking cytostatic drugs in initial stages of cancer could be classified as 5.x Single Dominant or Moderate Chronic Disease.

## Discussion

### Main findings

Throughout the study we assumed the perspective of an integrated healthcare provider. Within that context, obtaining individual costs linked to morbidity is important to deduce the related resource consumption profiles. According to the results, patients with different health conditions required a different effort from the health system. HIV, diabetes, heart failure, COPD, cystic fibrosis, rheumatoid arthritis, coronary insufficiency (and combinations of these) are examples of diseases which required a different balance of health resources. Moreover, some high-cost patients had clearly a Primary care profile, for example patients suffering heart failure with diabetes and COPD, whereas HIV patients for example, were treated mainly from hospital – Acute outpatient – services. Following this approach, the design of personalized care strategies would benefit from a more precise allocation of resources. On the other hand, from a healthcare planner’s perspective, the availability of precise information on resource consumption linked to morbidity may help to improve the state-of-the-art of incentive-based payment schemes for chronic care [31].

As an example of potential uses, since 2012 SSIBE has been developing a proactive and integrated care programme designed to adjust the use of health resources by patients with complex chronic diseases. Individuals are systematically identified as potential high consumers according to a predictive model based on prior use and morbidity, i.e. individual cost above the 95th percentile according to a logistic regression [15]. Individuals identified as potential high consumers are included in target lists, which are then delivered to the corresponding general practitioner [34]. Unfortunately, the model is unable to detail which specific costs and healthcare resources according to patient morbidity are required. Following the procedure presented, future revisions of the programme would include more precise interventions, tailored according to individual expected resource consumption, moving beyond the general scheme focused on restraining acute hospitalizations and accident & emergencies episodes from primary care.

A previous study fitted a set of different models to estimate drug consumption costs for the Baix Empordà population [32]. The authors used CRGs as a risk adjuster, obtaining a high predictive power compared to other models in the literature. Our results achieved a similar predictive power, but also included almost the whole range of healthcare services (NB: residential care was excluded). The original CRG articles as well as the recent research on predictive modeling [15,16,20,21] were focused on total healthcare costs. In contrast, our analysis improves insight into the specific healthcare costs and services required according to individual morbidity profiles.

The definition of healthcare services may vary across countries. For example, Acute outpatient may include a different composition of services. In fact, the service lines were defined according to the Catalan healthcare public system characteristics, but differed substantially from other studies, e.g. [13,14]. Moreover, the characteristics of SSIBE influenced the results of the analysis. It is important to remark that SSIBE is a healthcare provider responsible for providing the public first-level care assistance. Thus, complex care treatments and mental health were not included in costs. A previous study from Inoriza et al. established that External referrals represented 2.2% of the total episodes of care and 17% of the total costs for SSIBE in 2007 [33]. External referrals included high-complexity hospitalizations, psychiatric hospitalization and ambulatory mental health. Although the case presented may seem excessively restrictive, the algorithm is flexible enough to be used in a wide variety of situations, considering multidisciplinary organizations, different groups of services, processes, healthcare episodes or individuals. A fundamental idea throughout this work was to present a suitable method that is relatively easy to use and applicable to a wide variety of integrated care schemes.

### Modelling issues

Several articles considered a non-linear relationship between costs and age [4,13]. In general, healthcare costs and age tend to show a J-shaped curve association [35]. However, with the data set we analyzed, the linear assumption fits the data reasonably well because population is clustered in the central ages. Moreover, preliminary tests including squared and cubic age transformations failed to discern a clear impact on the costs of the different service lines and so were removed from the final specification of the models.

A prevalent estimator for individual healthcare expenditure and costs is the GLM-Gamma model. However, according to the literature, any GLM specification becomes inappropriate if the log-scale residuals have high kurtosis [4,6]. The Manning-Mullahy algorithm based on a choice between two classes of estimators: GLM and linear models on the log of costs, provides a suitable way to find a robust model. More complex specifications have been extensively discussed in more recent articles [9,11,12]. Among these, flexible link functions based on inverse Box-Cox transformations remain stable against various data problems and avoid potential bias caused by a wrong link assumption. Models based on flexible link functions provide a more general framework, including GLM with log link or the logistic regression as particular cases. However, from our point of view, the analysis proposed by Manning-Mullahy reaches a balanced compromise between simplicity and robustness.

### Limitations

As a first limitation of the study, it has to be said that this idea of simplicity, featured throughout the article, buckled when the output of the algorithm resulted in a heteroscedastic ols-model. In this case, the corrections required are difficult to implement and may consume a considerable amount of time. First, a robust estimation of standard errors for the regression coefficients should be used, otherwise confidence intervals and p-values can be incorrect. Throughout this article we have used the Huber-White estimate of the variance covariance matrix. Second, as mentioned earlier, the interpretation of the coefficients involved retransforming to the raw scale of the variable. We retransformed the coefficients using a secondary model for the variance, which included the log of the squared residuals as a dependent variable [5,29].

A second limitation concerns the specific results on patient profile results and the corresponding impact on the health system. Internal validity can be reasonably accepted, given that we included the whole population of interest – no selection bias was plausible – and adjusted for possible sources of confusion. However, in terms of external validity, it must be said that our specific results can only be directly extrapolated to populations with comparable demographic and morbidity characteristics and, moreover, under a comparable scheme of healthcare services provision.

Finally, the incompleteness of the residential care information must be considered as a significant limitation as well. SSIBE residential care services, when compared to healthcare, had less detailed information systems. In this regard, further research would be required to check and improve standards of social care information, especially concerning new forms of care delivery.

## Conclusions

The Manning-Mullahy algorithm identified a set of suitable cost models for the different groups of services integrated care requires. Such a procedure can be applicable to individual resource consumption or costs related to special combinations of healthcare services or innovative care processes. Besides including demographic variables, the individual burden of morbidity is essential when allocating appropriate resources to targeted individuals.

## References

[1] Evers S, Paulus A (2015). Health econòmics and integrated care: a growing and challenging relationship. Int J Integr Care.

[2] Paulus A, Van Raak A, Maarse H (2008). Is integrated nursing home care cheaper than traditional care? A cost comparison. International Journal of Nursing Studies.

[3] Vondeling H (2004). Economic evaluation of integrated care: an introduction. International Journal of Integrated Care.

[4] Mullahy J (1998). Much Ado about two: reconsidering retransformation and the two-part model in health economics. Journal of Health Economics.

[5] Manning WG (1998). The logged dependent variable, heteroscedasticity, and the retransformation problem. Journal of Health Economics.

[6] Manning WG, Mullahy J (2001). Estimating log models: to transform or not to transform?. Journal of Health Economics.

[7] Deb P, Burgess JF (2003). A Quasi-experimental Comparison of Econometric Models for Health Care Expenditures. http://econpapers.repec.org/scripts/redir.pf?u=http%3A%2F%2Fecon.hunter.cuny.edu%2Fwp-content%2Fuploads%2Fsites%2F6%2FRePEc%2Fpapers%2FHunterEconWP212.pdf;h=repec:htr:hcecon:212.

[8] Beeuwkes Buntin M, Zaslavsky AM (2004). Too much ado about two-part models and transformation? Comparing methods of modeling Medicare expenditures. Journal of Health Economics.

[9] Manning WG, Basu A, Mullahy J (2005). Generalized modeling approaches to risk adjustment of skewed outcomes data. Journal of Health Economics.

[10] Duan N, Manning WG, Morris CN, Newhouse JP (1982). A Comparison of Alternative Models for the Demand of Medical Care.

[11] Mihaylova B, Briggs A, O’Hagan A, Thompson SG (2011). Review of statistical methods for analysing healthcare resources and costs. Health Econ.

[12] Hill SC, Miller GE (2010). Health expenditure estimation and functional form: applications of the generalized gamma and extended estimating equations models. Health Econ.

[13] Kasteridis P, Street A, Dolman M, Gallier L, Hudson K, Martin J, Wyer I (2015). Who would most benefit from improved integrated care? Implementing an analytical strategy in South Somerset. Int J Integr Care.

[14] Orueta JF, García-Álvarez A, García-Goñi M, Paolucci F, Nuño-Solinís R (2014). Prevalence and Costs of Multimorbidity by Deprivation Levels in the Basque Country: A Population Based Study Using Health Administrative Databases. Plos One.

[15] Coderch J, Sánchez Pérez I, Ibern P, Carreras M, Pérez-Berruezo X, Inoriza JM (2014). [Predicting individual risk of high healthcare cost to identify complex chronic patients]. Gaceta Sanitaria.

[16] Chechulin Y, Nazerian A, Rais S, Malikov K (2014). Predicting patients with high risk of becoming high-cost healthcare users in Ontario (Canada). Healthc Policy.

[17] Carreras M, García-Goñi M, Ibern P, Coderch J, Vall-Llosera L, Inoriza JM (2011). Estimates of patient costs related with population morbidity: can indirect costs affect the results?. European Journal of Health Economics.

[18] Cots F, Chiarello P, Carreras M, Gonzalez JG, Heras D, de Imaña M, Vecina F, del Oro M, Vaamonde N (2012). Red Española de Costes Hospitalarios (RECH): bases para una gestión clínica basada en la evidencia. Gest y Eval Cost Sanit.

[19] Clinical Risk Grouping Software (2004). Definitions Manual.

[20] Hughes JS, Averill RF, Eisenhandler J, Goldfield NI, Muldoon J, Neff JM, Gay J (2004). Clinical Risk Groups (CRGs): A Classification System for Risk-Adjusted Capitation-Based Payment and Health Care Management. Medical Care.

[21] Neff JM, Sharp VL, Muldoon J, Graham J, Myers K (2004). Profile of Medical Charges for Children by Health Status and Severity Level in a Washington State Health Plan. Health Services Research.

[22] Inoriza JM, Coderch J, Carreras M, Vall-Llosera L, Ibern P, García-Goñi M, Lisbona JM (2009). Measurement of morbidity attended in an integrated health care organization. Gaceta Sanitaria.

[23] Park R (1966). Estimation with heteroscedastic error terms. Econometrica.

[24] Hosmer DW, Lemeshow S (1980). Goodness of fit tests for the multiple logistic regression model. Communications in Statistics.

[25] Swanson DA, Tayman J, Bryan TM (2011). MAPE-R: a rescaled measure of accuracy for cross-sectional subnational population forecasts. Journal of Population Research.

[26] van Veen SH, van Kleef RC, van de Ven WP, van Vliet RC (2015). Is there one measure-of-fit that fits all? A taxonomy and review of measures-of-fit for risk-equalization models. Medical Care Research and Review.

[27] Pregibon D (1980). Goodness of Link Tests for Generalized Linear Models. Applied Statistics.

[28] Breusch TS, Pagan AR (1979). A Simple Test for Heteroscedasticity and Random Coefficient Variation. Econometrica.

[29] Zhou X-H, Stroupe KT, Tierney WM (2001). Regression analysis of health care charges with heteroscedasticity. Journal of the Royal Statistical Society: Series C (Applied Statistics).

[30] R Core Team (2015). R: A language and environment for statistical computing. http://www.R-project.org.

[31] Tsiachristas A, Dikkers C, Boland MR, Rutten-van Molken MP (2013). Exploring payment schemes used to promote integrated chronic care in Europe. Health Policy.

[32] Garcí-Goñi M, Ibern P (2008). Predictability of drug expenditures: An application using morbidity data. Health Economics.

[33] Inoriza JM, Carreras M, Lisbona JM, Sánchez E, Coderch J, Ibern P (2010). La despesa sanitària poblacional segons la morbiditat atesa. Estudis d’Economia de la Salut.

[34] Pérez X, Pérez M, Inoriza JM, Ibáñez A, Sánchez I, Coderch J (2012). Strategy for proactive integrated care for high-risk, high-cost patients/Estrategia de atención proactiva integrada a pacientes en riesgo de alto consumo de recursos. Int J Integr Care.

[35] Yamamoto DH (2013). Health Care Costs-From Birth to Death.

